# *Nephrolepis cordifolia* Extracts Attenuate Calcium Oxalate Nephrolithiasis Through Redox Modulation and Suppression of Crystal Retention

**DOI:** 10.3390/antiox15101326

**Published:** 2026-10-14

**Authors:** Jianjun Guo, Liang Yao, Jiahao Liu, Huanhuan Liu, Qin Li, Zishan Li, Yinghao Li

**Affiliations:** 1 College of Agriculture, Anshun University, Anshun 561000, China; 2 Charles Institute of Dermatology, School of Medicine, University College Dublin, D04 V1W8 Dublin, Ireland; 3 School of Public Health, Anhui University of Science and Technology, Huainan 232001, China; 4 College of Veterinary Medicine, Hunan Agricultural University, Changsha 410128, China

**Keywords:** *Nephrolepis cordifolia*, natural antioxidants, calcium oxalate nephrolithiasis, oxidative stress, redox homeostasis, crystal retention, CD44, osteopontin

## Abstract

Calcium oxalate (CaOx) nephrolithiasis is closely associated with crystal-induced oxidative injury, impaired antioxidant defenses, and enhanced crystal retention in renal tissues. *Nephrolepis cordifolia* is a medicinal fern traditionally used for urinary tract-related disorders and edema, but its antioxidant-related protective activity against CaOx nephrolithiasis has not been systematically investigated. In this study, ethanol and water extracts of *Nephrolepis cordifolia* tubers were evaluated using CaOx crystallization assays, HEK293T cell-based oxidative stress assays, and a glyoxylate-induced mouse model. The extracts inhibited CaOx crystal growth and aggregation and shifted SEM-observed crystal morphology from calcium oxalate monohydrate-like aggregates toward less adhesive calcium oxalate dihydrate-like forms. FTIR spectra further indicated extract-associated changes in the structural and hydration characteristics of the resulting CaOx crystals. In cells, the extracts showed good cytocompatibility and reduced intracellular reactive oxygen species. In vivo, both extracts reduced renal crystal deposition and histopathological injury, restored antioxidant defenses, decreased malondialdehyde accumulation, downregulated CD44 and osteopontin expression, and improved serum and urinary biochemical abnormalities. The water extract generally produced stronger protection than the ethanol extract. LC–MS profiling of both ethanol and water extracts annotated diverse constituents, including flavonoid-related compounds, glycosides, phenolic constituents, terpenoid-related molecules, and lipid-associated components. These findings indicate that *Nephrolepis cordifolia* extracts mitigate CaOx nephrolithiasis partly by restoring redox homeostasis and limiting oxidative injury-associated crystal retention.

## 1. Introduction

Nephrolithiasis is a common urological disorder with a high recurrence rate, and calcium oxalate (CaOx) stones account for most cases [1,2,3,4,5]. CaOx occurs mainly as calcium oxalate monohydrate (COM) or calcium oxalate dihydrate (COD); COM crystals adhere more strongly to renal epithelial surfaces, whereas COD crystals are generally less adhesive and more readily excreted [6,7,8,9,10]. Current interventions remove existing stones but do not sufficiently prevent recurrence [3,11,12], highlighting the need for preventive strategies that target crystallization, renal epithelial injury, and crystal retention.

Traditional herbal medicines and dietary bioactives have long been investigated as complementary approaches for preventing stone formation. Increasing evidence indicates that plant-derived compounds can inhibit CaOx crystallization, modulate crystal morphology, reduce crystal aggregation, and alleviate crystal-induced oxidative and inflammatory injury in renal tissues [13,14,15]. *Quercus dentata* Thunb. leaf extract has been shown to suppress CaOx crystal aggregation, promote the transformation of COM-like crystals toward COD-like forms, and alleviate renal injury through modulation of the OPN/CD44 and NLRP3 pathways [8]. Degraded soybean polysaccharides have also been reported to inhibit CaOx crystal aggregation and promote the repair of oxidatively injured renal epithelial cells [16]. Recent studies further support the role of plant-derived antioxidants in CaOx nephrolithiasis. For example, *Rhus coriaria* fruit extract was recently shown to attenuate experimental CaOx nephrolithiasis by restoring antioxidant defenses, reducing lipid peroxidation, decreasing renal crystal deposition, and lowering OPN expression [17]. Recent reviews have likewise identified antioxidant and anti-inflammatory activities, together with interference with crystal formation and urinary risk factors, as recurrent mechanisms of traditional medicinal plants and phytotherapeutic preparations used for urolithiasis [18,19]. Together, these findings highlight the growing interest in multi-target phytotherapeutic strategies that simultaneously target crystallization, oxidative injury, inflammation, and crystal retention. Nevertheless, many traditional medicinal plants with long-standing folk use remain insufficiently studied, and their potential anti-nephrolithic activities require systematic evaluation.

*Nephrolepis cordifolia* (L.) C.Presl, commonly known as tuberous sword fern, is a perennial fern widely distributed in tropical and subtropical regions. The species has been documented in traditional medicine for conditions including urinary tract infections, edema, jaundice, rheumatism, fever, and cough [20,21,22]. Its reported use in urinary tract-related conditions and edema provides an ethnomedical rationale for investigating potential effects on urinary stone-associated processes, although a specific traditional indication for CaOx nephrolithiasis has not yet been firmly established. Phytochemical investigations of *Nephrolepis cordifolia* and related *Nephrolepis* species have reported flavonoids, phenolic compounds, polysaccharide-like constituents, terpenoids, and alkaloids, although the chemical composition of *Nephrolepis cordifolia* remains incompletely characterized [23,24]. These properties are relevant to CaOx stone development because crystallization, oxidative epithelial injury, inflammation, crystal retention, and disturbed urinary homeostasis are interconnected pathological events. Thus, systematic pharmacological evaluation of *Nephrolepis cordifolia* may clarify whether its traditional use is accompanied by experimentally demonstrable protection against processes relevant to nephrolithiasis.

**Scheme 1:**
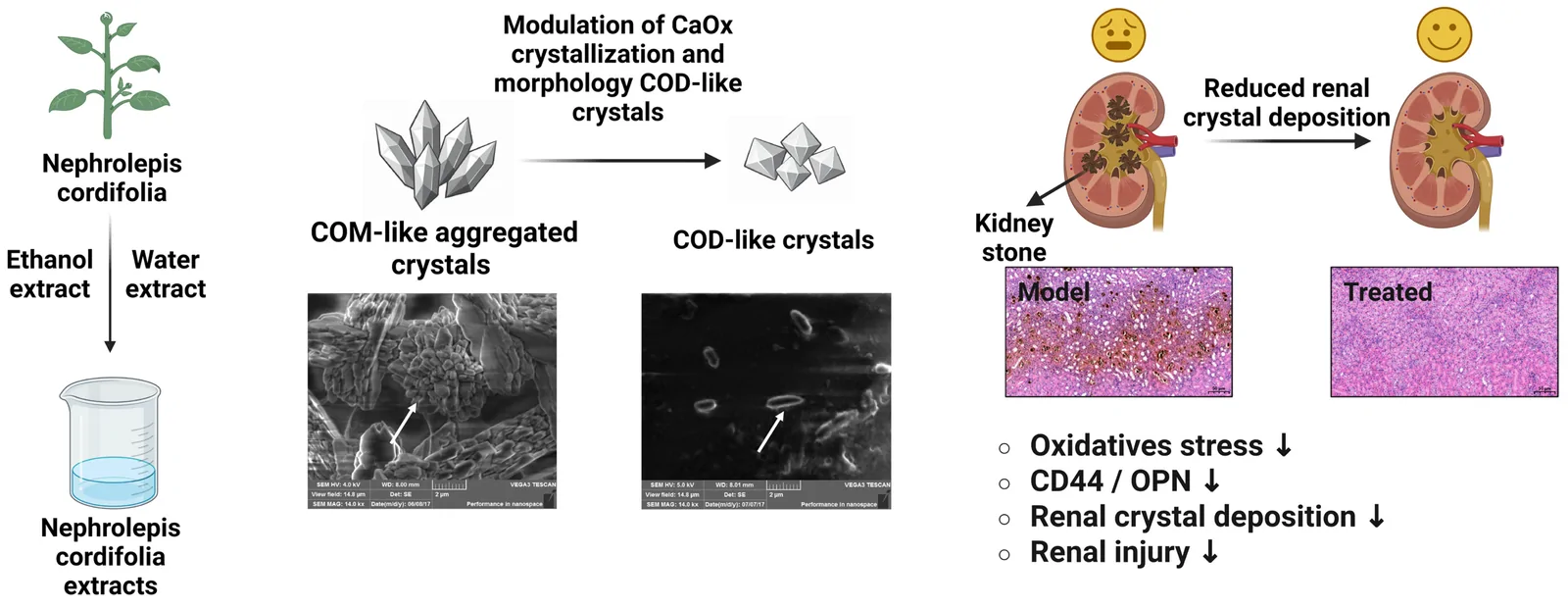
Proposed mechanisms of *Nephrolepis cordifolia* extracts against CaOx nephrolithiasis. The extracts regulate CaOx crystallization in vitro by shifting crystal morphology from COM-like aggregates toward COD-like forms and reducing aggregation and exert renoprotective effects in vivo by attenuating oxidative stress, suppressing CD44/OPN expression, and reducing crystal deposition. White arrows in the SEM images indicate representative CaOx crystals. Downward arrows denote decreases in the indicated parameters following extract treatment.

Based on this ethnopharmacological rationale, the present study evaluated the effects of *Nephrolepis cordifolia* tuber extracts on key physicochemical and biological events associated with CaOx nephrolithiasis (Scheme 1). Specifically, we examined CaOx crystallization and crystal morphology, cellular cytocompatibility and antioxidant effects, and preventive efficacy in a glyoxylate-induced mouse model. LC–MS profiling was additionally performed to provide an initial chemical framework for both the ethanol and water extracts. By integrating these complementary approaches, this study aimed to characterize the anti-nephrolithic potential of *Nephrolepis cordifolia* without attributing the observed effects to a single constituent or definitive molecular pathway.

## 2. Materials and Methods

### 2.1. Materials

*Nephrolepis cordifolia* tubers were collected in October 2024 from Anshun, Guizhou Province, China, and identified by Sisheng Sun, Associate Professor, Institute of Medicinal Plant Development, Chinese Academy of Medical Sciences and Peking Union Medical College. A voucher specimen (No. ASU-NC-GZ-20240301) was deposited at the College of Agriculture, Anshun University. Human embryonic kidney 293T (HEK293T) cells were obtained from the American Type Culture Collection (ATCC, Manassas, VA, USA; ATCC CRL-3216). Dulbecco’s Modified Eagle Medium (DMEM), fetal bovine serum (FBS), and penicillin–streptomycin were obtained from Gibco, Thermo Fisher Scientific (Waltham, MA, USA). Total Reactive Oxygen Species (ROS) Assay Kit 520 nm was purchased from Thermo Fisher Scientific (Waltham, MA, USA). Malondialdehyde (MDA), superoxide dismutase (SOD), glutathione (GSH), and catalase (CAT) assay kits were purchased from Wuhan Service Biotechnology Co., Ltd. (Wuhan, China). 

### 2.2. Preparation of Nephrolepis cordifolia Extracts

Fresh *Nephrolepis cordifolia* tubers were washed, air-dried, sliced, and dried in a hot-air oven at 60 °C before pulverization. For each extraction, 100 g of dried powder was macerated with 500 mL of either 60% ethanol or ultrapure water at room temperature for 7 days. The residue was re-extracted with a further 500 mL of the same solvent for another 7 days. The combined filtrates were concentrated to a crude drug-equivalent concentration of 0.5 g/mL, centrifuged at 10,000 rpm for 20 min, and stored at low temperature until use. Before experiments, extracts were diluted to the required concentrations with ultrapure water or culture medium as appropriate.

### 2.3. In Vitro Evaluation of CaOx Crystallization Modulation

#### 2.3.1. Establishment of the CaOx Crystallization System

CaOx crystallization was induced by mixing CaCl_2_ and Na_2_C_2_O_4_ to final ion concentrations of 0.8 mmol/L. Glass slides were incubated in the crystallization system with or without *Nephrolepis cordifolia* extracts (0.02, 0.1, or 0.5 g/mL crude drug-equivalent concentration) for 5 days. After air-drying, crystal morphology was examined by scanning electron microscopy (SEM; JSM-IT500HR, JEOL Ltd., Tokyo, Japan). Representative CaOx crystal samples generated without extract (NC) or in the presence of EE or WE were characterized using a Spotlight 400 Fourier-transform infrared (FTIR) spectrometer (PerkinElmer, Waltham, MA, USA) over the range of 4000–400 cm^−1^.

#### 2.3.2. Conductivity-Based Analysis of Crystallization Kinetics

Crystallization kinetics were assessed by conductivity monitoring [25]. CaCl_2_ and Na_2_C_2_O_4_ were mixed under stirring, and conductivity was recorded every 30 s in control solutions or in the presence of aqueous extract at 0.02, 0.1, or 0.5 g/mL.

### 2.4. Cell-Based Assays

#### 2.4.1. Cell Culture

Human embryonic kidney 293T (HEK293T) cells were obtained from the American Type Culture Collection (ATCC, Manassas, VA, USA; ATCC CRL-3216) and cultured in Dulbecco’s Modified Eagle Medium (DMEM, Gibco, Thermo Fisher Scientific, Waltham, MA, USA) supplemented with 10% fetal bovine serum (FBS, Gibco) and 1% penicillin–streptomycin (Gibco) at 37 °C in a humidified incubator with 5% CO_2_. Cells were passaged using trypsin and centrifuged at 1000 rpm for 4 min. For 96-well assays, cells were seeded at 30,000 cells/well. HEK293T cells were used as a model for cytocompatibility and oxidative-stress screening.

#### 2.4.2. Cell Cytotoxicity Assay

For cytotoxicity assays, HEK293T cells were seeded in 96-well plates at 30,000 cells/well and cultured for 24 h. Ethanol extract (EE) and water extract (WE) were prepared from a 10 mg/mL stock solution and diluted with complete DMEM to final concentrations of 0.0625–1.0 mg/mL. Cells were treated with 100 μL/well of each dilution for 24 h, washed with DPBS, and incubated with 100 μL of 10% Alamar Blue working solution for 1 h. Fluorescence was measured using a SpectraMax M3 microplate reader (Molecular Devices, San Jose, CA, USA) at excitation/emission wavelengths of 570/590 nm. Untreated cells were used as the 100% viability control.

#### 2.4.3. Intracellular Reactive Oxygen Species (ROS) Scavenging

For ROS analysis, HEK293T cells were seeded in 96-well plates at 30,000 cells/well and cultured for 24 h. Oxidative stress was induced with 100 μM H_2_O_2_ for 1 h. After removal of H_2_O_2_-containing medium, cells were treated with complete DMEM containing 1.0 mg/mL EE or WE for 1 h. The ROS probe was diluted 1:1000 with serum-free medium, added to the cells, and incubated for 30 min. Cells were washed with DPBS, and fluorescence was immediately measured using a SpectraMax M3 reader at excitation/emission wavelengths of 488/525 nm.

#### 2.4.4. Cell Scratch Assay

For scratch assays, HEK293T cells were seeded into 12-well plates at 1.5 × 10^6^ cells/well and cultured for 24 h until confluence. A linear scratch was made using a sterile 200 μL pipette tip, and wells were washed twice with DMEM to remove detached cells. Cells were then treated with 1.0 mg/mL EE or WE. Scratch widths were photographed at 0 and 24 h using an Olympus IX81 microscope (Olympus Corporation, Tokyo, Japan), and wound closure was calculated according to the following equation.

(1) $$\mathrm{Relative}\;\mathrm{migration}\;\mathrm{distance}\;(\%)\;=\left(\frac{d_{0}-d_{1}}{d_{0}}\right)\times 100$$

### 2.5. In Vivo Evaluation in a Glyoxylate-Induced Nephrolithiasis Mouse Model

#### 2.5.1. Glyoxylate Induced Nephrolithiasis Model and Treatment

Male SPF C57BL/6 mice were purchased from Chongqing Tengxin Biotechnology Co., Ltd. (Chongqing, China). A total of 24 male mice aged 8 weeks and weighing 19–23 g were housed two per cage under controlled conditions (25 ± 1 °C, 12 h light/dark cycle) with free access to food and water. After one week of acclimation, mice were randomly divided into four groups (*n* = 6): control (Ctrl), glyoxylate-induced nephrolithiasis model (Gly), glyoxylate plus ethanol extract (Gly+EE), and glyoxylate plus water extract (Gly+WE). All animal experiments were approved by the Animal Ethics Committee of Anshun University (Approval No. 2023-003; approval date: 21 June 2023) and were conducted in accordance with the relevant institutional guidelines and regulations.

EE and WE, each standardized to 0.5 g/mL crude drug-equivalent concentration, were administered by oral gavage twice daily for 14 consecutive days. The dosing volume was adjusted by body weight at 100 μL per 10 g per administration, equivalent to 10 mL/kg per administration and 5 g/kg crude drug equivalent per administration; the total daily dose was therefore 10 g/kg/day. This dose was expressed as a crude drug-equivalent dose rather than dried extract mass. Its selection was guided by dosing strategies used in previous phytopharmacological studies of glyoxylate-induced nephrolithiasis, in which relatively high oral doses of herbal preparations or human-to-mouse equivalent doses have been applied [26]. The Ctrl and Gly groups received an equal volume of vehicle. Nephrolithiasis was induced by intraperitoneal glyoxylate (100 mg/kg/day, 15 mg/mL in physiological saline, pH 7.4; injection volume 6.67 mL/kg) from day 8 to day 14. Extract gavage was performed from day 1 to day 14, so mice received extract alone during days 1–7 and extract plus glyoxylate challenge during days 8–14. Gavage was performed at 9:00 a.m. and 4:00 p.m. On day 15, mice were anesthetized, perfused, and euthanized, and kidneys were collected for analysis. Because extract administration was initiated before glyoxylate challenge, this experimental design was intended to evaluate preventive rather than therapeutic effects against nephrolithiasis.

#### 2.5.2. Histology and Immunohistochemistry

For histological examination [27], the left kidneys were fixed in 4% paraformaldehyde for 24 h and paraffin-embedded. Sections (4 μm) were cut and stained with hematoxylin and eosin (H&E) and von Kossa. Stained sections were observed using a microscope (CX43, Olympus, Tokyo, Japan). Calcium-containing crystal deposition in von Kossa-stained sections was quantified using ImageJ software (Version 1.54h; NIH, Bethesda, MD, USA).

#### 2.5.3. Oxidative Stress Analysis

Portions of the right kidney were homogenized in PBS using an ultrasonic processor. Oxidative stress markers, including malondialdehyde (MDA), superoxide dismutase (SOD), glutathione (GSH), and catalase (CAT), were measured according to the manufacturer’s instructions (Servicebio, Wuhan, China) [28].

#### 2.5.4. Serum Biochemical Analysis

Blood samples were collected from the retro-orbital sinus. After standing for 1 h, samples were centrifuged at 3000 rpm for 15 min. The serum was collected and stored at −80 °C. Before analysis, samples were thawed at once, and the levels of serum creatinine, blood urea nitrogen (BUN), calcium, phosphorus, magnesium, uric acid, alanine aminotransferase (ALT), and aspartate aminotransferase (AST) were determined using an automatic biochemical analyzer (Chemray 800, Shenzhen, China) [29].

#### 2.5.5. Urine Biochemical Analysis

The urine was collected from each group using metabolic cages after 24 h. Samples were centrifuged at 3000 rpm for 15 min, and the supernatants were stored at −80 °C. Prior to analysis, urine samples were thawed simultaneously, and urinary calcium, creatinine, phosphorus, and oxalate levels were measured using a urine analyzer (Urit-500B, Guilin, China) [30].

### 2.6. LC–MS-Based Chemical Profiling of Nephrolepis cordifolia Extracts

The EE and WE were subjected to LC–MS/MS analysis using a Xevo G2-XS QTOF system (Waters, Milford, MA, USA). Dried extract was dissolved in methanol, filtered through a 0.22 μm membrane, and analyzed by UPLC-QTOF-MS/MS. Chromatographic separation was performed on a reversed-phase C18 column (2.1 × 50 mm, 1.7 μm) at 40 °C. The mobile phases were 0.1% formic acid in water (A) and 0.1% formic acid in acetonitrile (B), with a flow rate of 0.4 mL/min. The gradient was 0–1 min, 5% B; 1–35 min, 5–98% B; 35–37 min, 98% B; and 37.1–40 min, re-equilibration to 5% B. ESI-QTOF detection was performed in positive and negative ion modes with capillary voltage 2.0 kV, source temperature 110 °C, desolvation temperature 400 °C, nitrogen desolvation gas flow 800 L/h, and mass range *m*/*z* 50–1200. MS/MS data were acquired in data-dependent mode with collision energy ramped from 20 to 40 eV. Tentative annotations were based on accurate mass, retention time, adduct ions, MS/MS fragmentation, database matching, and literature comparison.

### 2.7. Statistical Analysis

All quantitative data are presented as the mean ± standard deviation (SD). Data analysis was conducted using GraphPad Prism 10 software (GraphPad Software, Boston, MA, USA). ImageJ software (Version 1.54h; NIH, Bethesda, MD, USA) was used for image quantification. Normality was assessed using the Shapiro–Wilk test, and homogeneity of variances was assessed using the Brown–Forsythe test. Statistical comparisons among multiple groups were performed using ordinary one-way analysis of variance (ANOVA). Where applicable, multiple comparisons were performed using Tukey’s multiple comparisons test. A value of *p* < 0.05 was considered statistically significant.

## 3. Results and Discussion

### 3.1. In Vitro Effects of Nephrolepis cordifolia Extracts on CaC_2_O_4_ Crystallization

#### 3.1.1. Crystal Morphology by SEM

**Figure 1:**
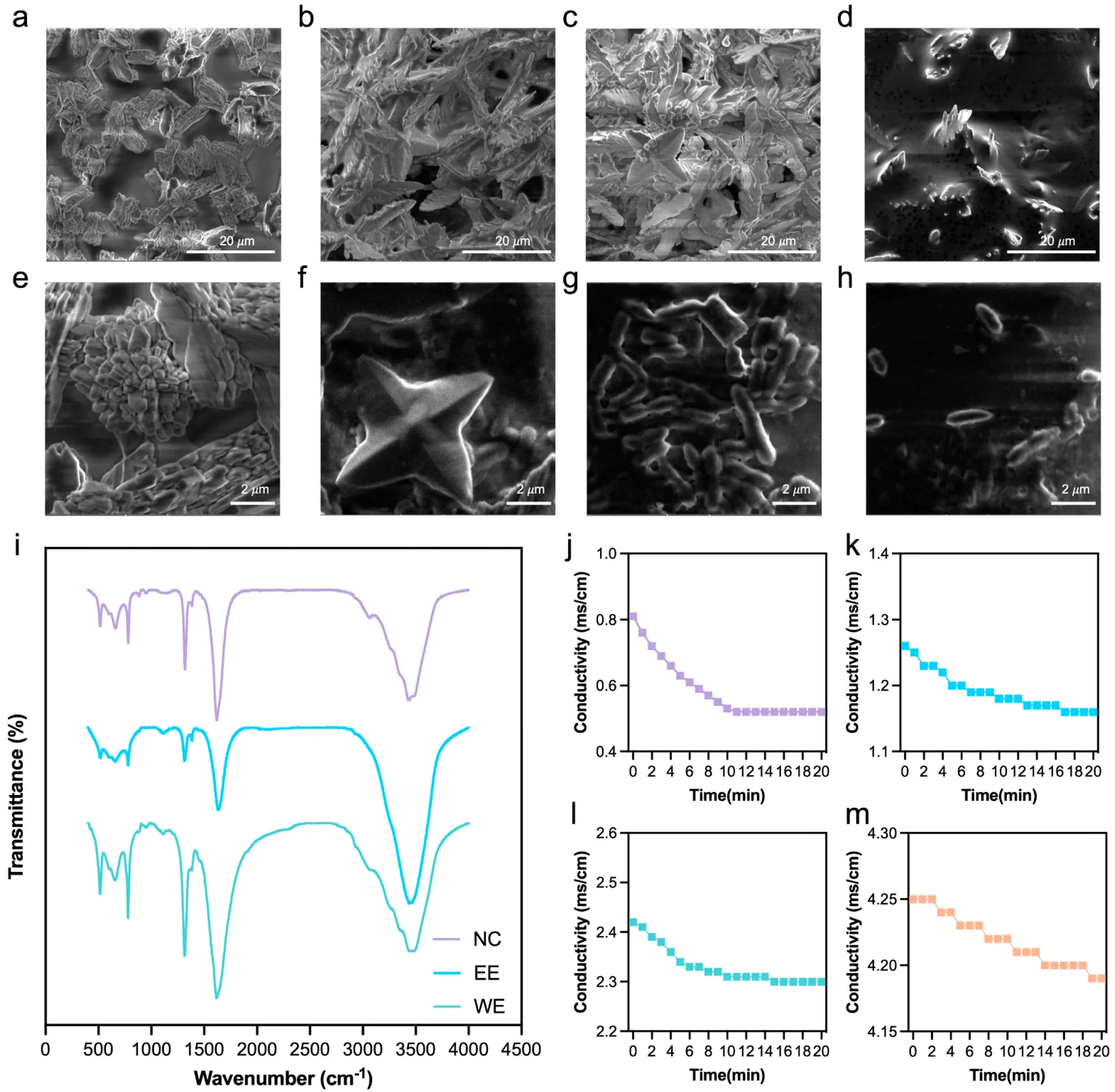
Effects of *Nephrolepis cordifolia* extracts on CaOx crystallization and growth kinetics. (**a**–**d**) EE: SEM images of CaOx crystals formed in the presence of different concentrations of *Nephrolepis cordifolia* EE: (**a**) control (without extract), (**b**) 0.02 g/mL, (**c**) 0.1 g/mL, and (**d**) 0.5 g/mL. (**e**–**h**) WE: SEM images of CaOx crystals formed in the presence of different concentrations of *Nephrolepis cordifolia* WE: (**e**) control (without extract), (**f**) 0.02 g/mL, (**g**) 0.1 g/mL, and (**h**) 0.5 g/mL. (**i**) FTIR spectra of CaOx crystals formed in the absence or presence of *Nephrolepis cordifolia* EE or WE. FTIR was used as qualitative supporting characterization and was not used for quantitative COM/COD phase assignment. (**j**–**m**) Conductivity kinetics in the presence of WE: Time-dependent changes in conductivity during CaOx crystal growth in the presence of *Nephrolepis cordifolia* WE: (**j**) pure water control, (**k**) 0.02 g/mL, (**l**) 0.1 g/mL, and (**m**) 0.5 g/mL.

Since CaOx (CaC_2_O_4_) is the predominant constituent of nephrolithiasis, its crystallization process, including nucleation, growth, and aggregation, is considered a critical event in nephrolithiasis [6,7,8,9,10]. To evaluate whether *Nephrolepis cordifolia* extracts could interfere with these processes, in vitro crystallization assays were performed. SEM was first used to examine changes in crystal morphology and abundance under different extract concentrations. In the control system, SEM showed mainly irregular COM-like aggregates. In the presence of *Nephrolepis cordifolia* EE, crystal aggregation decreased and the morphology shifted toward smaller, more dispersed COD-like crystals, especially at higher concentrations (Figure 1a–d). The WE produced a similar but generally stronger morphology-modulating effect (Figure 1e–h), with fewer large aggregates and more COD-like forms [8,16,31]. Because the present assignment is based primarily on SEM morphology, the crystal forms are described as COM-like or COD-like rather than definitively identified phases. These findings suggest that *Nephrolepis cordifolia* extracts interfere with CaOx growth and aggregation. The effect may be related to hydrophilic constituents, phenolics, flavonoids, or polysaccharide-like components capable of interacting with crystal surfaces.

#### 3.1.2. FTIR Characterization of CaOx Crystals

FTIR spectroscopy was used as a complementary method to characterize extract-associated changes in the spectral and hydration features of CaOx crystals [8,25]. All samples exhibited the principal absorption regions characteristic of CaOx crystals, including bands in the hydroxyl stretching region and prominent absorptions around 1600 cm^−1^ and within the fingerprint region (Figure 1i). Compared with the NC group, the EE- and WE-treated crystals showed changes in band shape and relative intensity, particularly around 1600 cm^−1^ and approximately 900–1200 cm^−1^. These spectral variations suggest that extract constituents may have altered the local bonding or hydration environment of the forming crystals. The changes were more evident in the WE group, consistent with the stronger morphology-modulating effects observed by SEM. Because the present FTIR data do not permit reliable quantitative phase analysis, no COM/COD ratio or phase fraction was inferred from the spectra. Accordingly, FTIR was treated only as qualitative supporting characterization, whereas interpretation of COM-like and COD-like forms was based primarily on SEM morphology.

#### 3.1.3. Crystallization Kinetics by Conductivity Analysis

During CaC_2_O_4_ crystallization, free Ca^2+^ and C_2_O_4_^2−^ ions are progressively consumed, resulting in a decrease in solution conductivity. Thus, real time conductivity monitoring provides a convenient method to evaluate the dynamic crystallization process, with the early conductivity decline mainly reflecting nucleation and the later stage corresponding to crystal growth and aggregation [32]. Conductivity monitoring further supported an inhibitory effect on CaOx crystallization. Control solutions showed a rapid conductivity decrease, consistent with ion consumption during crystal formation, whereas aqueous extract delayed and attenuated this decrease in a concentration-dependent manner (Figure 1j–m). At 0.5 g/mL, conductivity decreased more slowly and reached equilibrium later, indicating reduced CaOx nucleation and growth. These kinetic data are consistent with the SEM observations and support a crystallization-modulating role for the WE.

### 3.2. Nephrolepis cordifolia Exhibits Cytocompatibility and Antioxidant Activity In Vitro

#### 3.2.1. Cytocompatibility in HEK293T Cells

**Figure 2:**
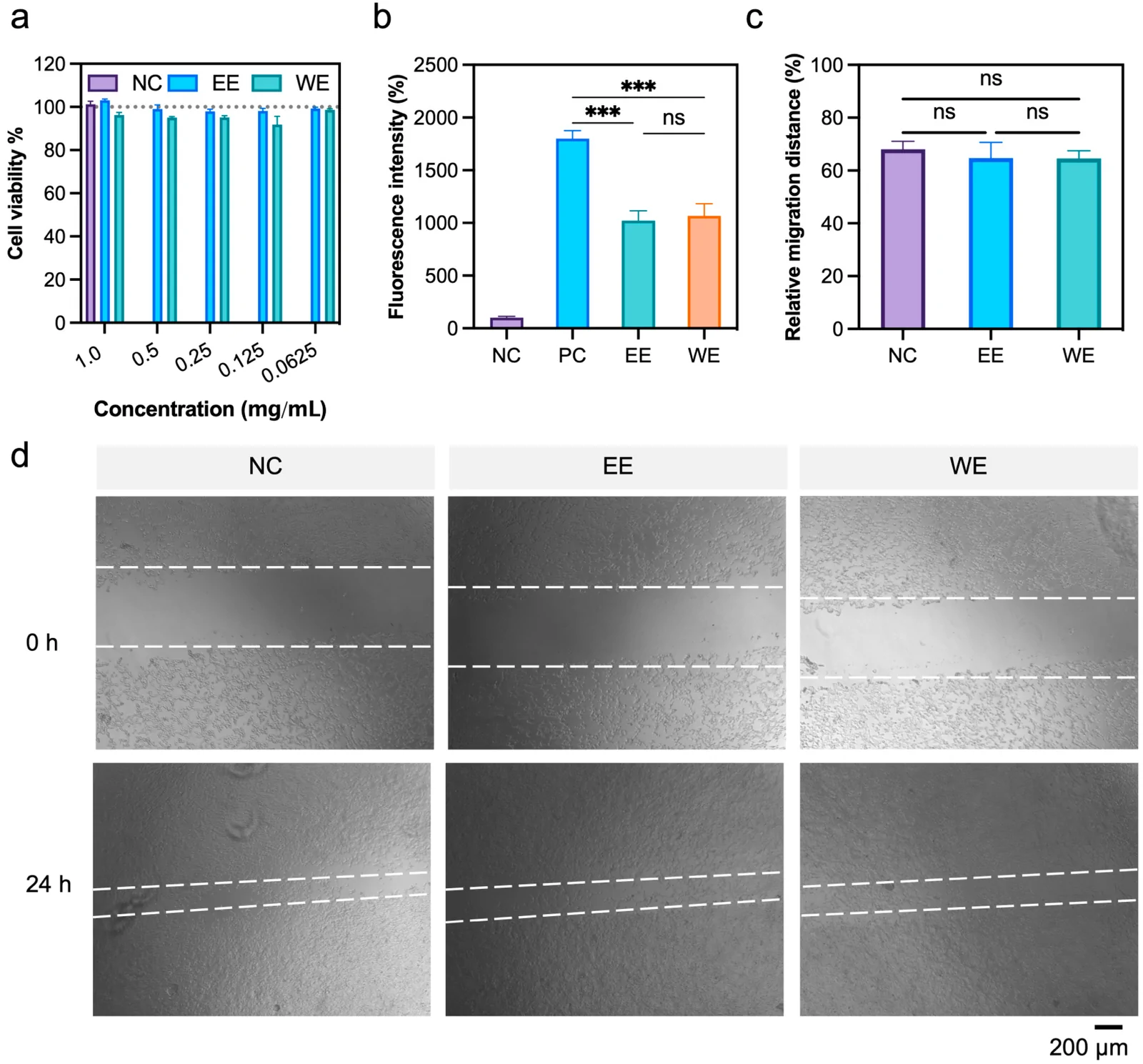
Cytocompatibility, antioxidant activity, and migration-related effects of *Nephrolepis cordifolia* extracts in HEK293T cells. (**a**) Cell viability of HEK293T cells after treatment with different concentrations of *Nephrolepis cordifolia* tuber EE and WE. (**b**) Effects of EE and WE on intracellular ROS levels in HEK293T cells under oxidative stress, with 100 μM H_2_O_2_ as the positive control. (**c**) Relative migration distance of HEK293T cells after treatment with different extracts. (**d**) Representative scratch images at 0 and 24 h. Data are presented as mean ± SD. Statistical significance was determined by one-way ANOVA followed by Tukey’s multiple comparisons test. *** *p* < 0.001; ns, not significant.

The biological effects of *Nephrolepis cordifolia* extracts were further evaluated in HEK293T cells. As shown in Figure 2a, both the EE and WE maintained cell viability above 90% over the tested concentration range of 0.0625–1.0 mg/mL, indicating good cytocompatibility in this preliminary cellular model.

#### 3.2.2. Reduction in Intracellular ROS

ROS overproduction is an important mediator of CaOx-associated renal injury [33,34]. To evaluate the antioxidant effects of the extracts at the cellular level, intracellular ROS levels were measured under H_2_O_2_-induced oxidative stress. Exposure to 100 μM H_2_O_2_ markedly increased ROS fluorescence, whereas treatment with either EE or WE at 1.0 mg/mL significantly reduced intracellular ROS accumulation (Figure 2b). No significant difference was observed between EE and WE, indicating that the two extracts exhibited comparable antioxidant effects in HEK293T cells. These findings demonstrate that both extracts effectively attenuated H_2_O_2_-induced intracellular oxidative stress.

#### 3.2.3. Preservation of Cell Scratch Closure Capacity

The effects of the extracts on epithelial repair capacity were further examined using a scratch assay. Representative images and quantitative analysis (Figure 2c,d) showed no significant differences in relative migration distance between extract-treated and control cells, indicating that neither EE nor WE impaired cell migration or closure capacity. Although no promotive effect on scratch closure was observed, the preservation of normal epithelial closure is still relevant because delayed epithelial repair after injury may increase exposure of damaged surfaces to CaOx crystals and facilitate crystal adhesion.

Collectively, these experiments demonstrate that *Nephrolepis cordifolia* extracts were cytocompatible, reduced intracellular oxidative stress, and did not impair scratch closure under the tested conditions. These cellular findings, together with the in vitro crystallization and in vivo results, support the protective potential of the extracts against biological events associated with CaOx nephrolithiasis.

### 3.3. Nephrolepis cordifolia Alleviates Glyoxylate-Induced Nephrolithiasis In Vivo

#### 3.3.1. Reduction in Renal Crystal Deposition and Histopathological Injury

Although *Nephrolepis cordifolia* extracts inhibited CaOx crystallization and reduced oxidative stress in vitro, in vivo evaluation is essential to determine whether these effects can be translated into a complex physiological environment. Unlike the in vitro crystallization system, nephrolithiasis in vivo involves crystal deposition, tubular epithelial injury, inflammation, and crystal retention within renal tissues. Therefore, a glyoxylate induced nephrolithiasis mouse model was established to further evaluate the preventive effects of *Nephrolepis cordifolia* extracts in vivo.

**Figure 3:**
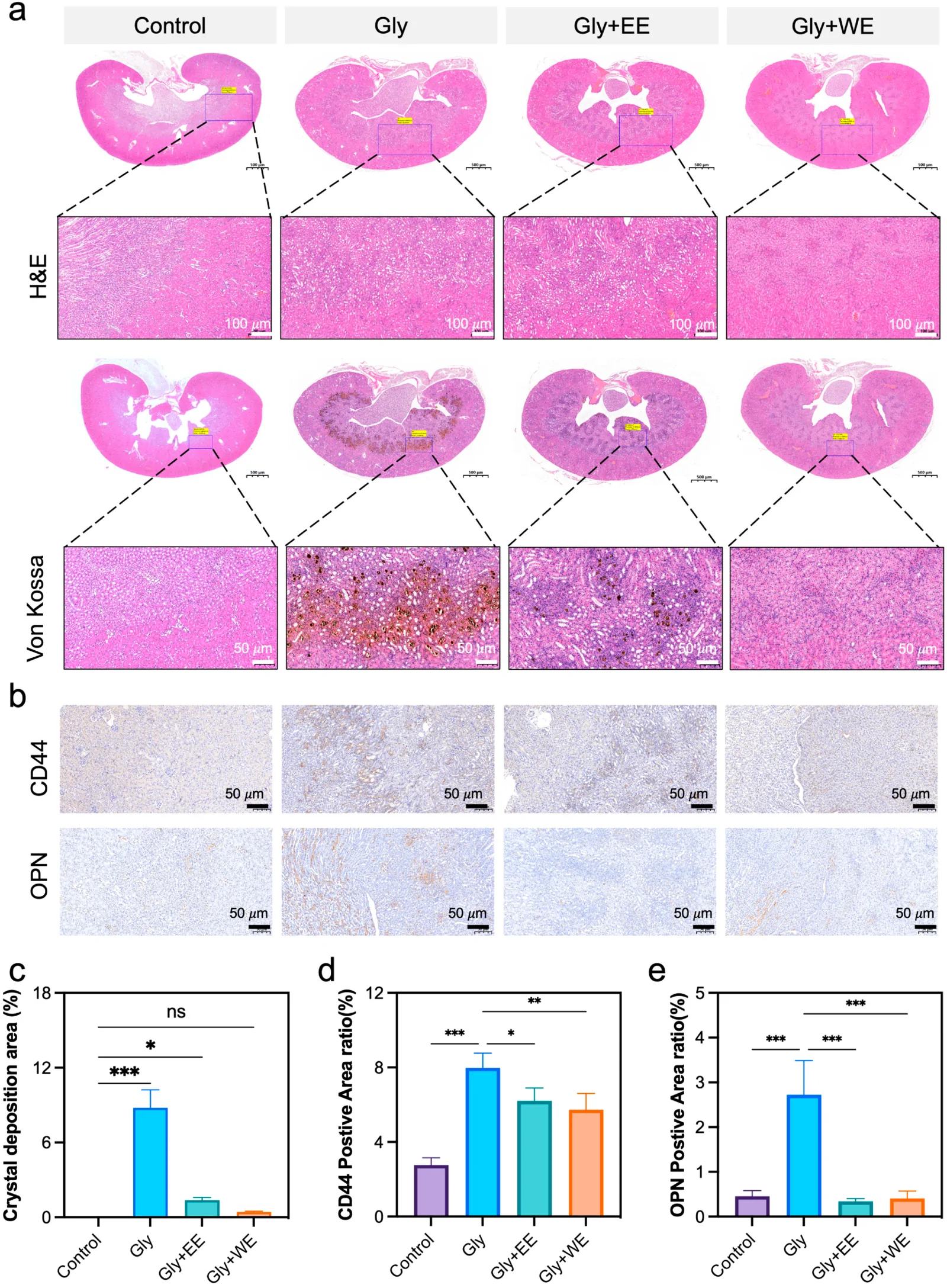
Effects of *Nephrolepis cordifolia* extracts on renal histopathology, crystal deposition, and expression of crystal adhesion-related markers in glyoxylate-induced nephrolithiasis mice. (**a**) Representative histological images of kidney sections stained with hematoxylin and eosin (H&E) and von Kossa. H&E staining shows renal tubular injury and structural alterations, while von Kossa staining indicates calcium deposition (black/brown areas). (**b**) Immunohistochemical staining of CD44 and osteopontin (OPN) in kidney tissues. (**c**) Quantitative analysis of the crystal deposition area in von Kossa-stained sections. (**d**,**e**) Semi-quantitative analysis of CD44-positive area (**d**) and OPN-positive area (**e**). Immunohistochemical staining was interpreted together with semi-quantitative image analysis of CD44- and OPN-positive areas. Mice were divided into control, glyoxylate-induced model (Gly), and treatment groups receiving ethanol extract (Gly+EE) or water extract (Gly+WE). Data are expressed as mean ± SD (*n* = 6). Statistical significance was determined by one-way ANOVA followed by Tukey’s multiple comparisons test. * *p* < 0.05, ** *p* < 0.01, *** *p* < 0.001; ns, not significant.

As shown in Figure 3a, control kidneys exhibited normal renal architecture, intact tubular morphology, and no obvious calcium deposition by von Kossa staining. In contrast, the Gly group showed typical pathological changes associated with nephrolithiasis, including tubular dilation, inflammatory cell infiltration, fibrotic changes, epithelial injury, and abundant black calcium deposits within tubular lumens, confirming successful model establishment (Figure 3a,c). Treatment with *Nephrolepis cordifolia* extracts markedly alleviated these changes. The WE strongly reduced tubular injury, inflammatory infiltration, fibrotic alterations, and renal calcium deposition, with renal structure largely preserved. The EE also decreased tissue injury and crystal deposition compared with the untreated Gly group, although residual pathological changes remained more evident than in the Gly+WE group. These results indicate that both extracts protected against glyoxylate-induced renal injury, with the WE showing a stronger effect on crystal deposition and tissue preservation.

To evaluate crystal retention-related changes, CD44 and osteopontin (OPN) were examined by immunohistochemistry. Both markers are associated with CaOx crystal adhesion and retention in renal tubular epithelial cells [8,35,36]. Strong CD44 and OPN immunoreactivity was observed in the Gly group, particularly in tubular regions with crystal deposition, whereas both extract-treated groups showed reduced staining intensity (Figure 3b,d,e). The decrease in CD44 and OPN expression was more pronounced in the Gly+WE group, consistent with its stronger inhibitory effect on renal crystal accumulation.

Taken together, these findings demonstrate that *Nephrolepis cordifolia* extracts, especially the WE, reduced renal crystal deposition, alleviated histopathological injury, and downregulated crystal retention-related markers in glyoxylate-induced nephrolithiasis mice.

#### 3.3.2. Attenuation of Oxidative Stress

**Figure 4:**
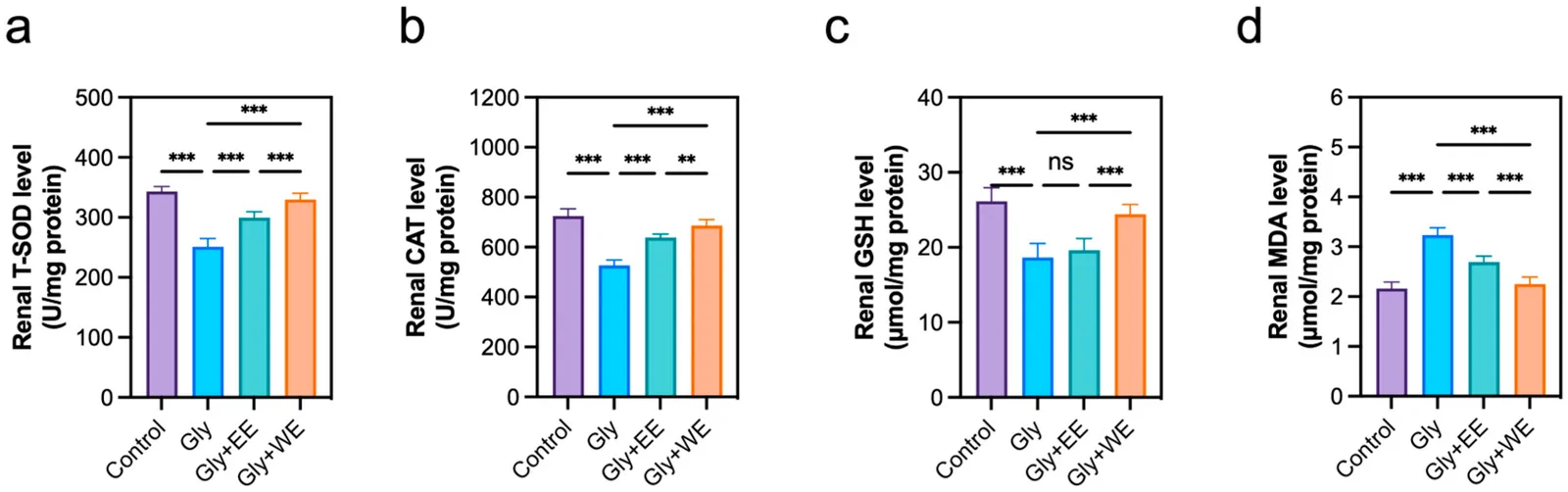
Effects of *Nephrolepis cordifolia* extracts on oxidative stress in glyoxylate-induced nephrolithiasis mice. (**a**–**d**) Oxidative stress indices in kidney tissues: superoxide dismutase (SOD, (**a**)), catalase (CAT, (**b**)), glutathione (GSH, (**c**)), and malondialdehyde (MDA, (**d**)). Data are expressed as mean ± SD (*n* = 6). Statistical significance was determined by one-way ANOVA followed by Tukey’s multiple comparisons test. ** *p* < 0.01, *** *p* < 0.001; ns, not significant.

Oxidative stress is a key contributor to nephrolithiasis-associated renal injury [27,37]. As shown in Figure 4, glyoxylate administration markedly disrupted renal redox homeostasis, as indicated by significant decreases in total superoxide dismutase (T-SOD), catalase (CAT), and glutathione (GSH), together with increased malondialdehyde (MDA), compared with the control group. These changes indicate impaired antioxidant defense and enhanced lipid peroxidation during glyoxylate-induced nephrolithiasis.

Treatment with *Nephrolepis cordifolia* extracts attenuated these oxidative alterations. Both Gly+EE and Gly+WE restored renal T-SOD and CAT levels and reduced MDA accumulation relative to the untreated Gly group. The WE showed a stronger overall antioxidant effect, particularly in restoring T-SOD and CAT activities and suppressing MDA elevation, whereas GSH recovery was relatively modest after extract treatment. These oxidative stress results are consistent with the histological and IHC findings. Oxidative injury can damage renal tubular epithelium and create a favorable surface for CaOx crystal adhesion and retention. Therefore, restoration of redox balance by *Nephrolepis cordifolia* extracts may limit epithelial injury and contribute to reduced CD44/OPN expression and decreased renal crystal deposition.

Overall, these findings indicate that *Nephrolepis cordifolia* extracts, particularly the WE, alleviated oxidative stress in glyoxylate-induced nephrolithiasis and may protect against crystal retention partly by attenuating crystal-induced oxidative injury.

#### 3.3.3. Improvement of Serum and Urinary Biochemical Abnormalities

To further determine whether the histological and molecular improvements were accompanied by functional recovery, serum and urinary biochemical parameters were evaluated. These indices reflect renal function, metabolic balance, and lithogenic risk during nephrolithiasis.

**Figure 5:**
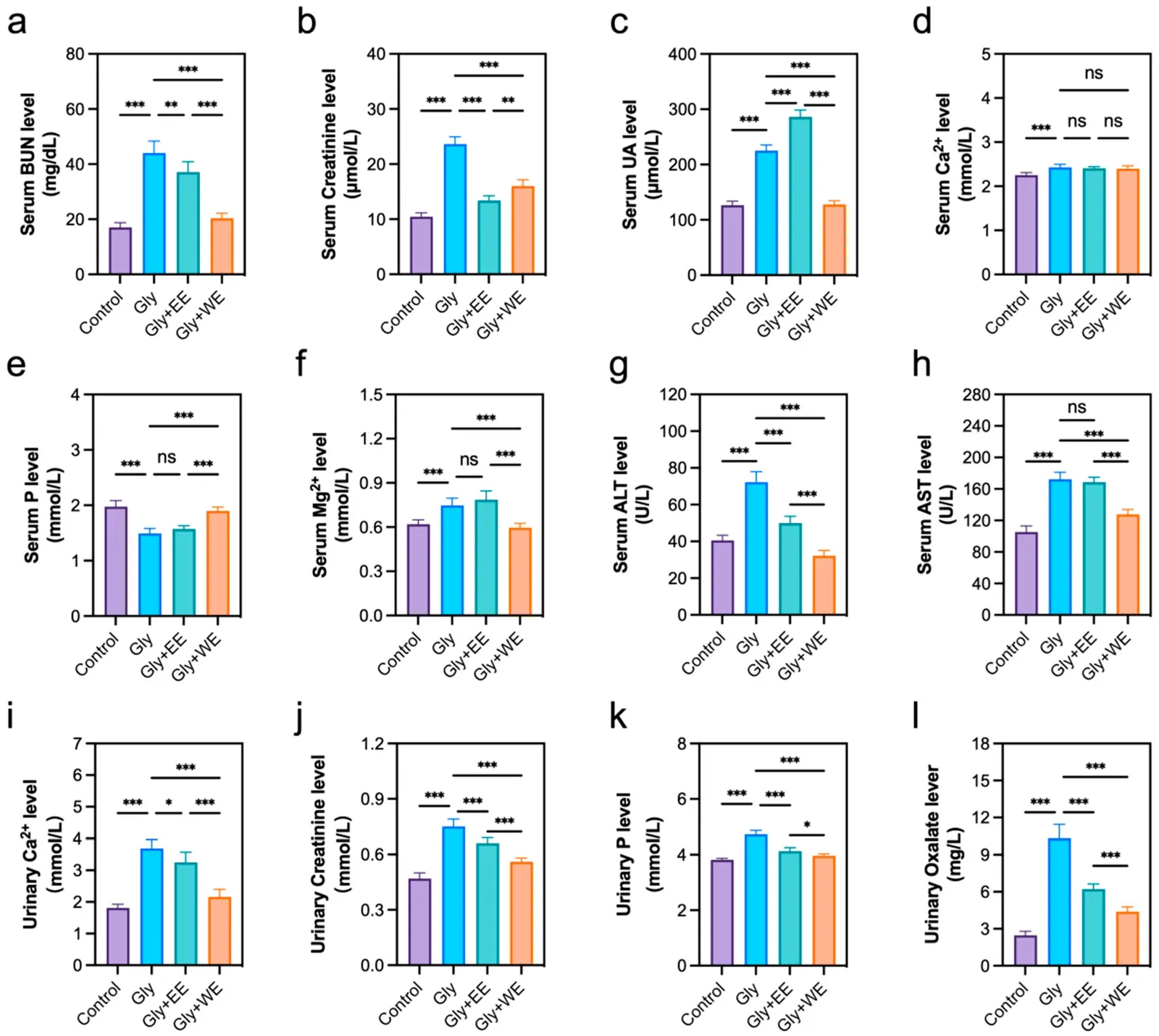
Effects of *Nephrolepis cordifolia* extracts on serum and urinary biochemical parameters in glyoxylate-induced nephrolithiasis mice. (**a**–**h**) Serum biochemical parameters: urea nitrogen (BUN, (**a**)), creatinine (**b**), uric acid (**c**), calcium (**d**), phosphorus (**e**), magnesium (Mg^2+^, (**f**)), alanine aminotransferase (ALT, (**g**)), and aspartate aminotransferase (AST, (**h**)). (**i**–**l**) Urinary biochemical parameters: calcium (**i**), creatinine (**j**), phosphorus (**k**), and oxalate (**l**). Mice were divided into control, glyoxylate-induced nephrolithiasis model (Gly), and treatment groups (Gly+EE, and Gly+WE). Data are expressed as mean ± SD (*n* = 6). Statistical significance was determined by one-way ANOVA followed by Tukey’s multiple comparisons test. * *p* < 0.05, ** *p* < 0.01, *** *p* < 0.001; ns, not significant.

Serum biochemical analysis revealed parameter-specific responses rather than uniform reductions across all indices. Glyoxylate administration markedly increased serum BUN, creatinine, uric acid, ALT, AST, and magnesium, whereas serum phosphorus decreased and serum calcium showed only a modest change. Both extracts reduced BUN and creatinine, with the WE generally producing a stronger restorative effect. The WE also markedly lowered serum uric acid, ALT, AST, and magnesium toward control levels. In contrast, the EE did not reduce uric acid relative to the Gly group and produced only limited improvements in ALT, AST, and magnesium. Neither extract significantly altered serum calcium compared with the Gly group. Serum phosphorus was restored toward the control level, particularly after water-extract treatment, indicating correction of glyoxylate-induced mineral imbalance rather than a generalized reduction in serum mineral concentrations (Figure 5a–h).

Glyoxylate exposure also increased urinary calcium, creatinine, phosphorus, and oxalate levels. Both EE and WE significantly reduced these urinary abnormalities, with the WE generally showing a stronger effect, particularly on urinary calcium, phosphorus, and oxalate (Figure 5i–l). These parameter-specific biochemical improvements support the protective effects of *N. cordifolia* extracts on renal function and the lithogenic urinary environment and further demonstrate the broader protective activity of the WE.

Potassium citrate is widely used clinically to reduce the recurrence risk of calcium-containing stones by increasing urinary citrate and alkalinizing the urine [3,30]. In contrast, the WE in the present study showed preventive effects across several experimental endpoints, including CaOx crystallization, renal oxidative stress, crystal deposition, and biochemical abnormalities. However, because potassium citrate was not included as a positive-control treatment, the relative efficacy of the WE and standard citrate therapy cannot be directly determined. Future studies incorporating potassium citrate as a comparator will be necessary to assess the translational relevance of the extract more rigorously.

### 3.4. Chemical Profiling of Nephrolepis cordifolia Extracts by LC–MS

**Figure 6:**
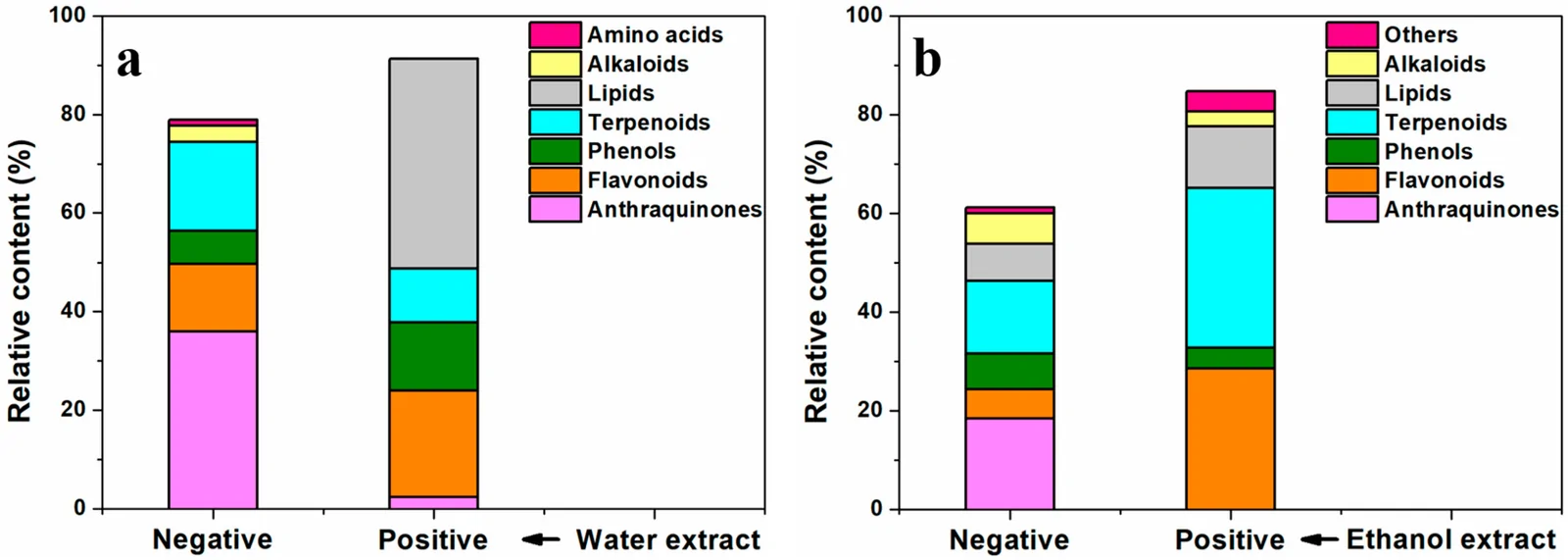
Relative abundance distributions of tentatively annotated phytochemical classes in *Nephrolepis cordifolia* extracts determined by LC–MS. (**a**) Water extract; (**b**) ethanol extract. Each panel shows the profiles obtained in negative and positive ion modes. Only tentatively annotated compounds with relative abundances >1% were included. Compounds were grouped by their phytochemical skeletons into anthraquinones, flavonoids, phenols, terpenoids, lipids, alkaloids, and amino acids, where applicable. “Others” represents included constituents not assigned to these classes. The percentages represent relative MS responses rather than absolute compound contents. Detailed compound annotations are provided in Tables S1–S4.

To provide a preliminary chemical basis for the observed anti-nephrolithic effects, both the EE and WE of *Nephrolepis cordifolia* were analyzed by LC–MS in positive and negative ion modes [38]. For comparative presentation, only tentatively annotated compounds with relative abundances greater than 1% were included in Tables S1–S4. The relative abundance distributions of the annotated phytochemical classes in the water and ethanol extracts are summarized in Figure 6a and Figure 6b, respectively.

The EE showed a chemically diverse profile containing flavonoid-related compounds, terpenoid-related constituents, glycosides, lipid-associated molecules, and other low- to medium-polarity metabolites. In the negative ion mode, the dominant constituent was 1,3-dihydroxy-2-hydroxymethylanthraquinone-3-O-β-D-xylopyranosyl-(1→6)-β-D-glucopyranoside (anthraquinones, 18.35%), together with 14-deoxyandrographolide-19β-glucoside (terpenoids, 11.20%), centaurein, kaempferol derivatives, daphnetin, and related constituents. In the positive ion mode, representative abundant constituents included Nelumboroside A (flavonoids, 13.15%), andrographolide, β-glyceryl stearate, methylophiopogonanone A, myricitrin, and several terpenoid-related compounds.

The WE displayed a distinct chemical profile. In the negative ion mode, 16 compounds with relative abundances above 1% accounted for 78.92% of the total relative abundance. Anthraquinones and anthraquinone glycosides represented the largest fraction (36.00%), followed by terpenoid/steroid glycosides (18.01%) and flavonoid glycosides (13.61%). The most abundant constituent was 1,3-dihydroxy-2-hydroxymethylanthraquinone-3-O-β-D-xylopyranosyl-(1→6)-β-D-glucopyranoside (anthraquinones, 30.24%). Phenolic acids and phenolic glycosides accounted for 6.83%, while alkaloid- and amino acid-related constituents were present at lower relative abundances. In the positive ion mode, 14 compounds with relative abundances above 1% accounted for 91.22% of the total relative abundance. Fatty acids, fatty acid esters, and glycerides were the predominant class (42.47%), followed by flavonoids and flavonoid glycosides (21.65%) and coumarins (13.73%). Major constituents included β-glyceryl stearate (17.57%), 5-methoxy-8-hydroxypsoralen (12.47%), bufotalin (10.99%), methyl pentadecanoate (10.86%), morin (10.73%), and glyceryl monopalmitate (9.78%). Overall, the WE of *Nephrolepis cordifolia* was mainly composed of anthraquinones, terpenoids, flavonoids and other glycosides in the negative ion mode and was rich in lipids, flavonoids and phenolic acids in the positive ion mode.

Anthraquinones, flavonoids, phenolic acids, terpenoids, glycosides, and lipid-related constituents were detected in the WE and 60% EE of *Nephrolepis cordifolia*, with glycoside-related constituents accounting for a relatively higher proportion in the WE. These distinct chemical profiles may contribute to the different biological activities observed for EE and WE. In particular, the relatively abundant anthraquinone-, flavonoid-, phenolic-, and glycoside-related constituents in the WE are consistent with compound classes that have been widely associated with antioxidant and cytoprotective activities. These constituents may therefore contribute, individually or collectively, to the reduction in oxidative stress observed in vivo.

The generally stronger protective activity of the WE may reflect the combined contribution of multiple hydrophilic glycosides, flavonoid-related constituents, phenolic acids, terpenoid-related compounds, and lipid-associated components. Nevertheless, the present LC–MS analysis provides only tentative compositional information and does not establish a causal relationship between any individual constituent and the observed biological effects.

### 3.5. Study Limitations

Several limitations of the present study should be acknowledged. First, the glyoxylate-induced nephrolithiasis model represents an acute and predominantly preventive experimental setting and therefore does not fully reproduce the chronic and recurrent nature of human calcium oxalate stone disease. Second, the crude drug-equivalent dose used in mice was relatively high, and its direct translation to a potential human dose is uncertain; therefore, dose–response studies, pharmacokinetic characterization, and appropriate human-equivalent dose estimation will be necessary before translational conclusions can be drawn. Third, HEK293T cells were used for preliminary cytocompatibility and oxidative-stress screening and do not fully recapitulate mature renal tubular epithelial physiology. Fourth, the reductions in CD44 and OPN expression were evaluated by immunohistochemistry without further validation by Western blotting or RT-qPCR. Finally, the LC–MS analysis provided tentative compositional information but did not establish causal relationships between individual constituents and the observed biological effects. Future studies should therefore incorporate more physiologically relevant tubular epithelial models, quantitative molecular validation, activity-guided fractionation, dose optimization, and longer-term in vivo models. To specifically evaluate therapeutic efficacy, future in vivo studies should initiate extract administration only after CaOx deposition has been established and longitudinally assess whether pre-existing crystal deposits are reduced or resolved during treatment.

## 4. Conclusions

In this study, authenticated *Nephrolepis cordifolia* tuber extracts showed protective effects against CaOx nephrolithiasis through complementary physicochemical and biological actions. In vitro, the extracts inhibited CaOx crystal growth and shifted the SEM-observed morphology from COM-like aggregates toward less adhesive COD-like forms. In vivo, they reduced renal crystal deposition, alleviated histopathological injury, improved serum and urinary biochemical abnormalities, attenuated oxidative stress, and lowered CD44 and OPN expression. The water extract generally exhibited stronger protection than the ethanol extract, whereas LC–MS profiling further revealed distinct chemical compositions of the ethanol and water extracts, providing an initial chemical basis for their observed activities. Overall, these findings provide experimental support for further ethnopharmacological investigation of *Nephrolepis cordifolia* in urinary tract-related disorders and identify CaOx nephrolithiasis as a potential preventive application.

## Data Availability

The original contributions presented in this study are included in the article/Supplementary Materials. Further inquiries can be directed to the corresponding authors.

## Supplementary Materials

The following supporting information can be downloaded at: https://www.mdpi.com/article/10.3390/antiox15101326/s1, Table S1. Putative identification of selected components in the ethanol extract of *Nephrolepis cordifolia* detected in positive ion mode based on LC–MS analysis. Table S2. Putative identification of selected components in the ethanol extract of *Nephrolepis cordifolia* detected in negative ion mode based on LC–MS analysis. Table S3. Putative identification of selected components in the water extract of *Nephrolepis cordifolia* detected in positive ion mode based on LC–MS analysis. Table S4. Putative identification of selected components in the water extract of *Nephrolepis cordifolia* detected in negative ion mode based on LC–MS analysis.

## Abbreviations

The following abbreviations are used in this manuscript:

ALT	Alanine aminotransferase
AST	Aspartate aminotransferase
BUN	Blood urea nitrogen
CAT	Catalase
CaOx	Calcium oxalate
COD	Calcium oxalate dihydrate
COM	Calcium oxalate monohydrate
EE	Ethanol extract
GSH	Glutathione
H&E	Hematoxylin and eosin
LC–MS	Liquid chromatography–mass spectrometry
MDA	Malondialdehyde
OPN	Osteopontin
ROS	Reactive oxygen species
SEM	Scanning electron microscopy
T-SOD	Total superoxide dismutase
WE	Water extract
