# Supplementary material for: Nephrolepis cordifolia Extracts Attenuate Calcium Oxalate Nephrolithiasis Through Redox Modulation and Suppression of Crystal Retention
Source: Antioxidants (Basel). 2026 Oct 14;15(10):1326. doi: 10.3390/antiox15101326 (PMC13701195; doi:10.3390/antiox15101326)
Supplement: Supplementary file 1 [file antioxidants-15-01326-s001.zip › antioxidants-4582445-supplementary.pdf]

SI

Nephrolepis cordifolia Extracts Attenuate Calcium Oxalate Nephrolithiasis through Redox Modulation and Suppression of Crystal Retention

Jianjun Guo [a, †, *], Liang Yao [b, †], Jiahao Liu [c], Qin Li [a], Huanhuan Liu [d], Zishan Li [b], Yinghao Li [b, *]

[a] College of Agriculture, Anshun University, Anshun 561000, P. R. China

[b] Charles Institute of Dermatology, School of Medicine, University College Dublin, Dublin D04 V1W8, Ireland

[c] School of Public Health, Anhui University of Science and Technology, Huainan 232001, China

[d] College of Veterinary Medicine, Hunan Agricultural University, Changsha 410128, China

* Correspondence: Jianjun Guo (jianjunguo868@163.com); Yinghao Li (yl2193@cam.ac.uk);

† These authors contributed equally to this work.

Table S1. Putative identification of selected components in the ethanol extract of Nephrolepis cordifolia detected in positive ion mode based on LC-MS analysis.

Group	Compound name	Molecular formula	Observed retention time (min)	Observed m/z	Response	Mass error (ppm)	Adduct (ion species)	Percentage
1	Nelumboroside A	$C_{27}H_{30}O_{16}$	4.95	611.1616	256256	1.6	+H, +Na, +K	13.15%
2	*β*-Glyceryl stearate	$C_{21}H_{42}O_4$	26.6	381.2943	147827	-8.4	+Na	7.19%
3	Methylophiopogonanone A	$C_{19}H_{18}O_6$	0.45	381.0759	124613	6.3	+K, +Na	6.06%
4	Methyl (24Z)-27-hydroxy-3-oxo-7,24-euphadien-21-oate	$C_{31}H_{48}O_4$	31.34	485.3584	100603	-8.6	+H	6.42%
5	Pyropheophorbide A	$C_{33}H_{34}N_4O_3$	25.44	535.2693	82069	-2	+H, +Na, -e	3.99%
6	Myricitrin	$C_{21}H_{20}O_{12}$	4.94	465.1007	81870	-4.5	+H, +Na, +K	6.50%
7	Asterinin E	$C_{26}H_{35}N_5O_9$	19.22	579.2813	68685	6.8	0	3.34%
8	SoyasaponinI_1	$C_{40}H_{75}NO_9$	30.92	736.5361	68285	3.7	+Na, +H, +K	3.32%

9	Andrographolide	C26H40O8	29.27	481.2821	64900	5.3	+H	8.30%
10	Azedarachin C	C32H42O10	24.07	609.2705	60577	5.7	+Na	3.83%
11	Centaurein	C21H20O11	5.85	449.1053	59297	-5.6	+H, +Na, +K	2.88%
12	Olibanumols I	C29H48O2	31.74	429.3695	54288	-7.5	+H	4.84%
13	xanthophyll	C40H56O2	29.15	568.4253	8282	-3.8	-e	2.00%
14	Ent-eudesmane-2α,4β,11-triol 11-O-β-D-glucopyranoside	C21H38O8	8.23	441.2434	46122	-5.6	+Na, +NH4, +K	2.24%
15	4-Hydroxyphenethyl triacontanoate	C38H68O3	35.56	595.5061	42165	0.1	+Na, +H, +K	2.35%
16	Methyl pentadecanoate	C16H32O2	22.33	279.2269	40337	-9.3	+Na	1.96%
17	(-)-Betulin	C7H14O6	3.85	212.1121	36821	-3.7	+NH4, +H	1.79%
18	Lysicamine	C18H13NO3	0.53	330.0542	34592	4.5	+K, +Na	1.68%
19	Barley alkali B	C29H40N8O5	8.97	619.2760	12759	1.1	+K	1.34%
20	Bufotalinin	C24H30O6	14.82	437.1904	30470	-7	+Na, +H, +NH4,	1.48%

Table S2. Putative identification of selected components in the ethanol extract of Nephrolepis cordifolia detected in negative ion mode based on LC-MS analysis.

Group	Compound name	Molecular formula	Observed retention time (min)	Observed m/z	Response	Mass error (ppm)	Adduct (ion species)	Percentage
1	1,3-Dihydroxy-2-hydroxymethylanthraquinone-3-O -β-D-xylopyranosyl-(1→6)-β-D-glucopyranoside	C26H28O14	4.94	609.1469	862333	1.3	+HCOO	18.35%
2	Umbelliferone	C9H6O3	3.95	161.0244	17924	-0.2	-H	1.31%
3	l-Pentadecene	C15H30	37.53	255.2329	7762	-0.3	+HCOO	1.54%
4	Ephedrine B	C29H38N4O5	20.02	581.3005	249839	4.2	+CH3COO	6.12%
5	Soyasphingosine I	C41H77NO9	32.81	786.5742	204115	0.7	+CH3COO	4.60%
6	Centaurin	C21H20O11	5.87	447.0939	198494	1.3	- H, + Cl	4.48%
7	Hemerolignanol A	C20H18O8	6.71	431.0986	102394	0.5	+HCOO	2.18%
8	Kaempferol-7-O-α-L-arabinofuranoside	C20H18O10	5.11	463.0888	72086	1.3	+HCOO	1.53%
9	Ent-eudesmane-2α,4β,11-triol	C21H38O8	8.23	463.255	63944	0.3	+HCOO, -	1.53%

	11-O-β-D-glucopyranoside						H, +Cl	
10	Soyasphingosine I_1	C40H75NO9	30.88	758.5422	61412	-0.2	+HCOO, -H,+Cl	1.31%
11	14-Deoxyandrographolide-19β-glucoside	C26H40O9	18.83	555.2845	55293	6.2	+CH3COO	11.20%
12	Daphnetin	C9H6O4	4.21	237.0407	51997	1.1	+CH3COO	2.42%
13	Cinnamaldehyde	C9H8O	2.52	177.0557	50797	-0.2	+HCOO, -H	1.24%
14	Lourutherin	C13H14O3S2	1.63	281.0302	49869	-3.6	-H	1.18%
15	20(R)-Ginsenoside Rh2	C36H62O8	29.11	621.436	49779	-2	-H	1.06%
16	Bruceoside F	C20H28O10	5.12	473.1665	48801	0.1	+HCOO,-H	1.04%

Table S3. Putative identification of selected components in the water extract of Nephrolepis cordifolia detected in positive ion mode based on LC–MS analysis.

Group	Compound name	Molecular formula	Observed retention time (min)	Observed m/z	Response	Mass error (ppm)	Adduct (ion species)	Percentage
1	β-Glyceryl stearate	C21H42O4	28.09	359.3158	2211	0.7	+H	17.57%
2	5-Methoxy-8-hydroxypsoralen	C11H6O5	0.51	219.0279	65685	−3.9	+H	12.47%
3	Bufotalin	C24H30O6	15.64	415.2122	57906	1.7	+H, +Na, +NH4	10.99%
4	Methyl pentadecanoate	C16H32O2	14.05	274.2757	57228	5.9	0	10.86%
5	Morin	C15H10O7	8.30	303.0512	5011	4.1	+H	10.73%
6	Glyceryl monopalmitate	C19H38O4	25.43	331.2854	51523	3.5	+H, +Na	9.78%
7	Nelumboroside A	C27H30O16	5.40	611.1601	40321	−1	+H	7.65%
8	Catenarin	C15H10O6	7.23	287.0564	12544	4.8	+H	2.38%
9	Octadecatetraenoic acid	C18H28O2	17.60	277.2171	5213	3.1	+H	1.82%
10	Cyanidin glycoside	C21H20O11	6.08	449.108	2028	0.4	+H	1.67%

11	Myricitrin	C21H20O12	5.44	465.1026	8446	−0.3	+H	1.60%
12	Monopalmitin	C20H40O4	14.38	362.3266	6720	0.3	0	1.28%
13	Umbelliferone	C9H6O3	3.07	163.0404	2691	8.9	+H	1.26%
14	cis-9-Octadecenal	C18H34O	28.56	267.2692	6086	3.6	+H	1.16%

Table S4. Putative identification of selected components in the water extract of Nephrolepis cordifolia detected in negative ion mode based on LC–MS analysis.

Group	Compound name	Molecular formula	Observed retention time (min)	Observed m/z	Response	Mass error (ppm)	Adduct (ion species)	Percentage
1	1,3-Dihydroxy-2-hydroxymethylanthraquinone-3-O-β-D-xylopyranosyl-(1→6)-β-D-glucopyranoside	$C_{26}H_{28}O_{14}$	3.92	609.147	5571	1.4	+HCOO, −H	30.24%
2	Ent-eudesmane-2α,4β,11-triol 11-O-β-D-glucopyranoside	$C_{21}H_{38}O_8$	9.08	463.2538	14605	−2.3	+HCOO, −H	8.20%
3	Cyanidin glycoside	$C_{21}H_{20}O_{11}$	6.37	447.0926	131905	−1.6	−H	7.74%
4	6-Hydroxyluteolin-7-β-D-glucoside	$C_{21}H_{20}O_{12}$	5.55	463.0872	84704	−2.2	−H	4.83%
5	2-Deoxy-20-hydroxyecdysone-3-β-D-glucopyranoside	$C_{33}H_{54}O_{11}$	8.99	671.3679	4054	4.6	+HCOO	3.80%
6	1,3,6-Trihydroxy-2-methylanthraquinone-3-O-β-D-glucopyranoside	$C_{21}H_{20}O_{10}$	7.22	431.0964	66093	−4.7	−H, +HCOO	3.77%
7	Bisdemethoxycurcumin	$C_{19}H_{16}O_4$	1.11	353.1048	29455	5	+HCOO	3.41%
8	Anonaine	$C_{18}H_{13}NO_3$	1.3	290.0835	41909	4.2	−H	3.33%

9	Turpinionosides A	C19H34O8	6.33	435.2221	39220	−3.4	+HCOO, +CH3COO	2.94%
10	Chicoric acid	C22H18O12	3.16	519.0783	32070	0.6	+HCOO, −H	2.17%
11	Anthraquinone	C14H8O2	1.14	207.0455	11826	1.8	−H	1.99%
12	Actinidioionoside	C19H34O9	2.99	451.2163	5567	−4.9	+HCOO	1.79%
13	Blumenol C glucoside	C19H32O7	8.99	431.2265	4446	−4.9	+CH3COO	1.28%
14	2-Methoxy-4-acetylphenol 1-O-α-L-rhamnopyranosyl-(1″→6′)-β-D-glucopyranoside	C21H30O12	5.55	473.1655	19704	−2	−H	1.25%
15	Asparagine	C4H8N2O3	3.05	177.0501	19953	−9.3	+HCOO	1.14%
16	Puerarin apioside	C26H28O13	5.79	593.1528	9669	2.8	+HCOO	1.04%
